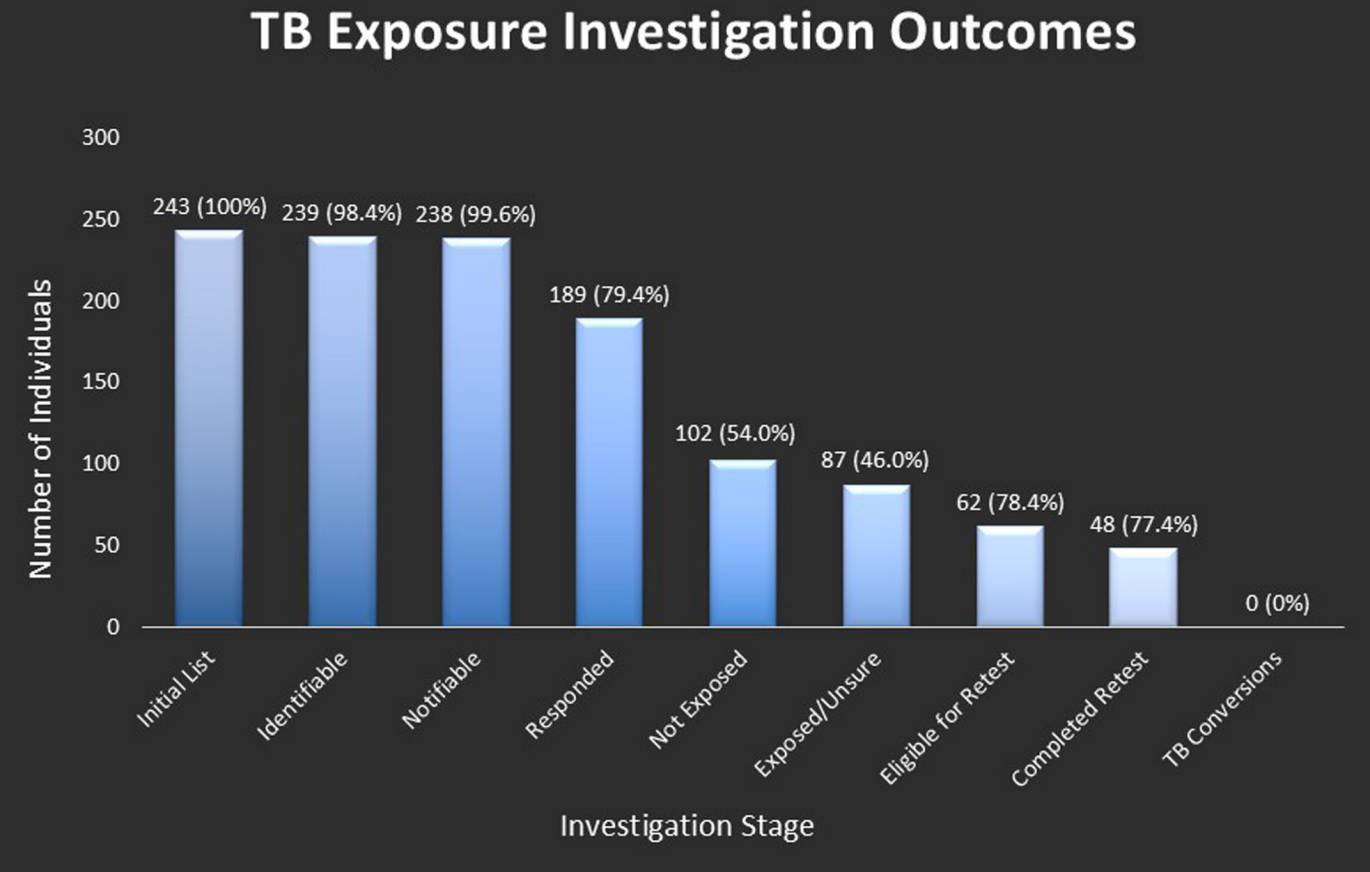# 321 Development and Implementation of “Isolation Precaution Stewardship Rounds”: A Pilot Study in a Children’s Hospital

**DOI:** 10.1017/ash.2026.10668

**Published:** 2026-06-23

**Authors:** Jeremy Berger, Gregory Zilli, Virginia Caples, Lou Ann Bruno-Murtha, Kevan Lutchman, Jenna Cherenzia, Eirini Iliaki

**Affiliations:** 1 Cambridge Health Alliance, Harvard School of Public Health; 2 Cambridge Health Alliance; 3 Cambridge Health Alliance, Harvard Medical School

## Abstract

**Background:** After three decades of declining tuberculosis (TB) incidence in the United States, case counts have increased annually since 2020, including an 8% rise in 2024. Undiagnosed TB poses a risk in healthcare settings because its clinical overlap with other respiratory illnesses can delay diagnosis and expose unprotected healthcare workers (HCWs) to active TB during routine job duties. Despite this risk, there is no standardized CDC definition of TB exposure for HCWs, resulting in variability in exposure investigations, notification, and follow-up. Safety-net hospitals such as Cambridge Health Alliance (CHA), a 300-bed health system, are increasingly affected by complex exposure events. Over the past year, seven patients with active pulmonary TB potentially exposed nearly 250 staff across six clinical areas, requiring multiple notification rounds despite prompt protocols, highlighting gaps in follow-up and uncertainty about actionable exposure definitions. Objective: Using the definition of employee exposure as ?15 minutes within 6 feet of a source case without mask/respirator, this study aimed to evaluate the effectiveness of post-exposure investigation protocols, determine TB screening test conversion rates among exposed employees, and assess the incidence of active TB disease during the study period. **Methods:** We reviewed email outreach to HCWs, employee communications, and TB testing records, and compared institutional practices with CDC and National Tuberculosis Controllers Association (NTCA) guidance. Analysis focused on accurate identification of exposed employees and completion of recommended follow-up testing using IGRA or TST. **Results:** Key barriers included incomplete exposure lists, fragmented interdepartmental communication, and inconsistent documentation of TB history,necessitating additional outreach efforts and contributing to a more time intensive notification process. The audit identified 243 potentially exposed staff, of whom 238 were contacted. 189 (79.4%) responded; non-responders included former employees, staff on leave, and those who did not reply. Among respondents, 87 (46%) met exposure criteria. Twenty-five (28.7%) had prior positive TB tests and were excluded from retesting. Of the 62 eligible for follow-up testing, 48 (77.4%) completed testing. No new TB infections or active TB cases were identified. **Conclusions:** Despite logistical challenges, the investigation achieved a high response rate with no new TB cases detected. Our study identified opportunities for improvement in data management, communication, and exposure classification. Standardizing TB exposure definitions and implementing centralized tracking systems may improve efficiency, consistency, and HCW protection. These findings support the effectiveness of current infection prevention measures while underscoring the need for a standardized national or regional approach.

**Figure:**
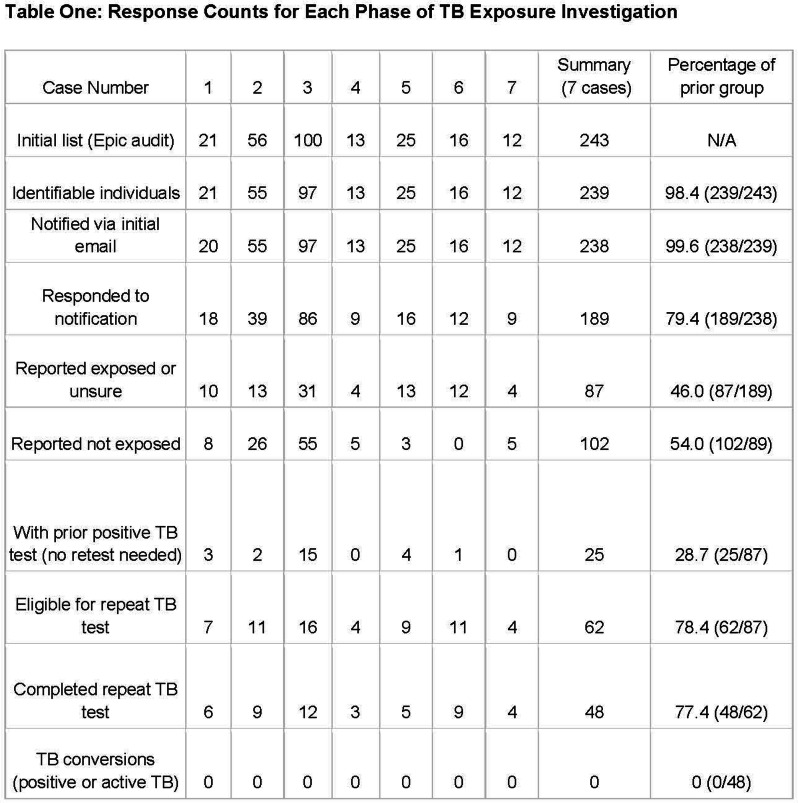

**Figure:**
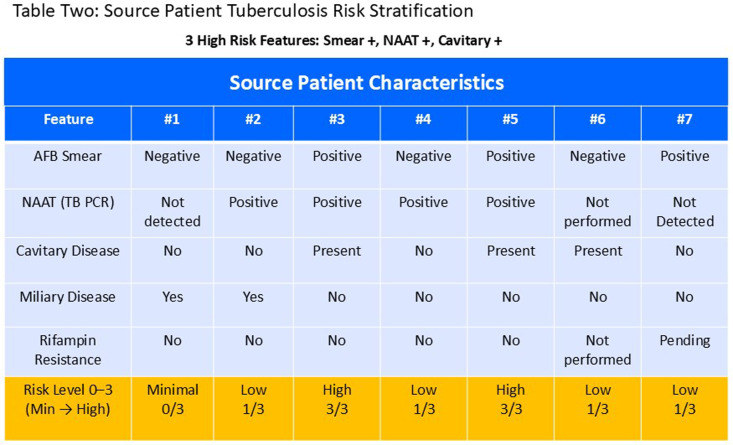

**Figure:**